# Targeting Neuropilin-1 to Inhibit VEGF Signaling in Cancer: Comparison of Therapeutic Approaches

**DOI:** 10.1371/journal.pcbi.0020180

**Published:** 2006-12-29

**Authors:** Feilim Mac Gabhann, Aleksander S Popel

**Affiliations:** Department of Biomedical Engineering, Johns Hopkins University School of Medicine, Baltimore, Maryland, United States of America; Harvard University, United States of America

## Abstract

Angiogenesis (neovascularization) plays a crucial role in a variety of physiological and pathological conditions including cancer, cardiovascular disease, and wound healing. Vascular endothelial growth factor (VEGF) is a critical regulator of angiogenesis. Multiple VEGF receptors are expressed on endothelial cells, including signaling receptor tyrosine kinases (VEGFR1 and VEGFR2) and the nonsignaling co-receptor Neuropilin-1. Neuropilin-1 binds only the isoform of VEGF responsible for pathological angiogenesis (VEGF_165_), and is thus a potential target for inhibiting VEGF signaling. Using the first molecularly detailed computational model of VEGF and its receptors, we have shown previously that the VEGFR–Neuropilin interactions explain the observed differential effects of VEGF isoforms on VEGF signaling in vitro, and demonstrated potent VEGF inhibition by an antibody to Neuropilin-1 that does not block ligand binding but blocks subsequent receptor coupling. In the present study, we extend that computational model to simulation of in vivo VEGF transport and binding, and predict the in vivo efficacy of several Neuropilin-targeted therapies in inhibiting VEGF signaling: (a) blocking Neuropilin-1 expression; (b) blocking VEGF binding to Neuropilin-1; (c) blocking Neuropilin–VEGFR coupling. The model predicts that blockade of Neuropilin–VEGFR coupling is significantly more effective than other approaches in decreasing VEGF–VEGFR2 signaling. In addition, tumor types with different receptor expression levels respond differently to each of these treatments. In designing human therapeutics, the mechanism of attacking the target plays a significant role in the outcome: of the strategies tested here, drugs with similar properties to the Neuropilin-1 antibody are predicted to be most effective. The tumor type and the microenvironment of the target tissue are also significant in determining therapeutic efficacy of each of the treatments studied.

## Synopsis

Neuropilin is a co-receptor for some of the isoforms of the vascular endothelial growth factor (VEGF) family. The presence of Neuropilin on endothelial or other cells increases binding of these isoforms to their signaling receptor VEGFR2, thus increasing pro-angiogenesis signaling and stimulating vascular growth. Neuropilin is thus a suitable target for anti-angiogenesis therapy, which holds promise for the treatment of vasculature-dependent diseases such as cancer and diabetic retinopathy. In this study, Mac Gabhann and Popel perform computational simulations of VEGF transport in breast cancer, using a previously validated model of VEGF–VEGF receptor interactions, as well as geometrical information on the tumor itself—tumor cells, vasculature, and extracellular matrix. Three different molecular therapies targeting Neuropilin are tested in silico, and the simulations predict that one of these therapies will be effective at reducing VEGFR2 signaling in certain types (or subtypes) of tumors, while the others will not. Thus, we demonstrate that identification of a target molecule is not sufficient; different therapeutic strategies targeting the same molecule may result in different outcomes.

## Introduction

Angiogenesis (neovascularization), the growth of new blood microvessels from preexisting microvasculature, is a critical physiological process for the growth of developing organs and during wound healing, ovulation, and pregnancy. Coronary or peripheral ischemia may be relieved by inducing angiogenesis [1,2], while diseases of hypervascularization, such as cancer or diabetic retinopathy, are targets of anti-angiogenic drugs [3,4]. Neuronal expression of angiogenic receptors [5,6] suggests that this work may also be relevant to the development nervous system. Our goal is to propose effective targeted therapies using anatomically accurate and molecularly detailed computational models of the growth factors and receptors involved in angiogenesis. In this study, we predict that three methods of targeting the same molecule (Neuropilin-1) result in distinct therapeutic outcomes, and that one of these methods is more effective (in terms of decreasing VEGF–VEGFR2 signaling for a defined period of time following treatment) than the others. Thus, identification of a therapeutic target must be followed by rational design of the targeting molecule to obtain characteristics that maximize the therapeutic potential. In addition, the microenvironment in which the drug is to act—for example, the expression level of receptors in the tissue—is a critical factor in the impact of the therapy.

Vascular endothelial growth factor (VEGF) is a family of secreted glycoproteins and critical regulators of angiogenesis [7,8]. In vitro, VEGF increases endothelial cell survival, proliferation, and migration. In vivo, it increases vascular permeability, activates endothelial cells, and acts as a chemoattractant for nascent vessel sprouts. Multiple splice isoforms of VEGF exist; the two most abundant in the human are VEGF_121_ and VEGF_165_. Both isoforms bind to the VEGF receptor tyrosine kinases (VEGFRs) to induce signals. VEGF_165_ also interacts with nonsignaling Neuropilin co-receptors and with proteoglycans of the extracellular matrix (ECM) [9,10] (Figure 1). The binding sites on VEGF_165_ for VEGFR2 and Neuropilin-1 are nonoverlapping, so VEGF_165_ may bind both simultaneously [9]. There are thus two parallel pathways for VEGF_165_ to bind its signaling receptor: binding directly to VEGFR2; and binding to Neuropilin-1, which presents VEGF to VEGFR2 (coupling the two receptors together). VEGF_121_ can only form VEGFR2 complexes directly [10]. The VEGF_165_–Neuropilin interaction is thus of particular value as a therapeutic target because VEGF_165_ is the isoform of VEGF that has been identified as inducing pathological angiogenesis [11,12]: aberrant angiogenic signaling may be targeted while allowing the normal levels of physiological VEGF signaling to continue.

**Figure 1:**
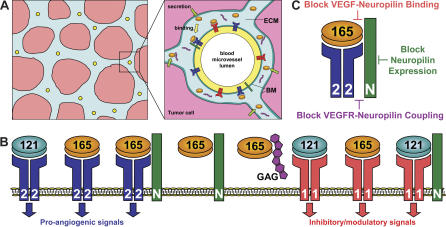
Schematics of VEGF Transport in Tumors, VEGF Receptor Binding, and Therapeutic Strategies (A) Schematic of the in vivo model. Parenchymal cells secrete VEGF; VEGF_121_ is freely diffusible, but VEGF_165_ can be sequestered by proteoglycans in the ECM (light gray) and the basement membranes (dark gray). The isoforms bind to VEGF receptors on the endothelial cells. (B) VEGF isoforms bind to VEGFR2 that transduces the angiogenic signal intracellularly. VEGF_121_ does not bind Neuropilin-1; VEGF_165_ may bind both VEGFR2 and Neuropilin-1 simultaneously. Thus there are two pathways for the binding of VEGF_165_ to the signaling VEGFR2 receptor: first by binding directly, and second by binding Neuropilin-1 and then diffusing laterally on the cell surface to couple to VEGFR2. VEGFR1, which modulates the signaling of VEGFR2, binds both isoforms of VEGF. VEGFR1 also binds directly to Neuropilin-1. This complex is permissive for VEGF_121_–VEGFR1 binding but not VEGF_165_–VEGFR1; thus, high levels of Neuropilin-1 displace VEGF_165_ from VEGFR1, making it available for VEGFR2 binding. Only VEGF_165_ binds directly to the ECM binding site (represented by GAG chains). (C) By targeting Neuropilin-1, we can target specifically VEGF_165_-induced signaling. Three methods for targeting Neuropilin-1 are analyzed here: blockade of Neuropilin-1 expression (e.g., using siRNA); blockade of VEGF–Neuropilin binding (e.g., using a fragment of placental growth factor to occupy the binding site); and blockade of VEGFR–Neuropilin coupling (e.g., using an antibody for Neuropilin-1 that does not interfere with VEGF–Neuropilin binding).

In previous work [9,13], we developed computational models of VEGF interactions with endothelial cell receptors in vitro, and incorporated previously published experimental data to estimate the kinetic rate of VEGFR2-Neuropilin coupling by VEGF_165_. We showed that VEGFR2–Neuropilin coupling is sufficient to account for the observed differential effects of VEGF isoforms on multiple cell types and that our model reproduces the distinct VEGF binding and signaling effects on each of these cell types [10,14–16]. In addition, we used the model to distinguish between alternate hypotheses of molecular mechanisms of action and demonstrated that the Neuropilin-1 antibody under investigation acts by blocking VEGFR–Neuropilin coupling, not by blocking VEGF–Neuropilin binding. Here, we extend that validated model of the molecular interactions of the VEGF family and its receptors to predict the in vivo behavior of the system by including the ECM and basement membranes, as well as multiple cell types (tumor cells and endothelial cells) and geometrical parameters characteristic of the tissue.

Three methods for targeting the VEGF_165_–Neuropilin interaction are modeled here. First, a blockade of Neuropilin-1 expression may be induced by use of siRNA or other methods to prevent the synthesis of the protein in the cells. Second, a protein that occupies the VEGF binding site on Neuropilin-1 can compete with VEGF_165_ for binding to that receptor. An example is a fragment of the placental growth factor isoform PlGF_2_. Full-length PlGF_2_ (a VEGF homolog) binds Neuropilin-1 and VEGFR1 (but not VEGFR2) [17]. The fragment, denoted PlGF_2Δ_ here, contains only the Neuropilin-1 binding site and does not bind to VEGFR1. This protein has been used to block VEGF binding to Neuropilin in vitro [14,17]. An alternative would be a Neuropilin-binding fragment of VEGF_165_ itself [18,19]. Third, we may block the interaction between Neuropilins and the VEGFRs, preventing the presentation of VEGF_165_ to the signaling receptor, but permitting Neuropilin to sequester that isoform. This may be done using a Neuropilin-1 antibody [20–23] that we have previously characterized as permitting VEGF binding to Neuropilin-1, but blocking the subsequent VEGFR coupling [9]. Each of these three strategies has been demonstrated to inhibit VEGF signaling in in vitro assays [14,15,17,24]; here we predict their in vivo efficacy.

This is the first computational model to our knowledge to include the interactions of the VEGF family and their receptors explicitly and in biophysical detail. The model includes the kinetics of all ligand–receptor interactions, which allows us to examine both short-term and long-term behavior of the system. All the parameters for the model have been obtained from previously published experimental data. Analysis of characteristic parameters shows that the kinetics of VEGF interactions are slower than the diffusion process, so diffusion is assumed to be fast, and we construct a compartmental model (i.e., spatial gradients of VEGF are not considered) with parenchymal cells secreting VEGF into the interstitial space and VEGF binding to receptors on the endothelial cell surface (Figure 1A).

The geometrical parameters of the tissue under investigation here (breast tumor) are also incorporated into the model: interstitial space, tumor cell volume and surface area, microvessel volume and surface area. Changes to these parameters would result in changes to the kinetic parameters and concentrations in the model. The results presented here are therefore tissue-specific, but the model may be applied to other tissues.

VEGFR2 is the primary signaling receptor for VEGF, and we first analyze the results of the model for a tissue in which the endothelial cells express VEGFR2 and Neuropilin-1, but not VEGFR1; the effect of VEGFR1 is considered later. Initially, the system is in a steady state, as VEGF is secreted by the parenchymal cells and internalized by the endothelial cells, resulting in a flux through the interstitial space and the ECM (Figure 1A). One of three treatments is initiated at time zero and the time course of VEGF binding followed for 48 hours. VEGF–VEGFR2 and VEGF–VEGFR1 binding are taken as a surrogate for VEGF signaling.

## Results

### Development of the Computational Model

#### Computational model.

We constructed a computational model of VEGF transport and interactions with its receptors in tumor tissue in vivo. The interstitial space between the tumor cells and the blood vessels is divided into three regions: the ECM, and the two basement membranes surrounding the tumor cells and the blood vessel endothelial cells (TBM and EBM, respectively). VEGF is secreted by the tumor cells and binds to cell surface receptors on the endothelial cells. In the case of VEGF_165_, it may also be sequestered by VEGF binding sites in the ECM and basement membranes. This sequestered VEGF can serve as a reservoir to buffer dynamic changes in free VEGF concentration. The binding interactions between VEGF_121_, VEGF_165,_ and the receptors VEGFR1, VEGFR2, and Neuropilin-1 are shown in Figure 2A and 2B. We assume that the concentration of free (unbound) VEGF is uniform across the interstitial space. The Damkohler number (the ratio of diffusion time to reaction time) is significantly less than one for this tissue, indicating that diffusion is significantly faster than the kinetics of binding to and unbinding from ECM glycosaminoglycan (GAG) chains or cell surface receptors. The possible formation of diffusion-limited VEGF gradients will therefore not be studied here; instead, our model represents average VEGF in the interstitium, and average receptor binding on the cell surface. Spatial variability in binding due to gradient formation may also be important for angiogenic signaling in vivo [25,26], but is not dealt with here; the variability would likely impact each of the three therapeutic strategies under consideration similarly. The mean concentration values in spatial models are predicted to be the same as the concentration in spatially averaged models such as this [25,27]. Although we do not model gradients here, there are major spatial features—basement membrane, and cell surfaces—that have a significant impact on VEGF distribution and VEGF receptor signaling. Thus the system is described by a set of coupled nonlinear ordinary differential equations. For interstitial proteins, these equations are as follows:

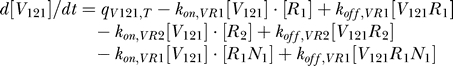


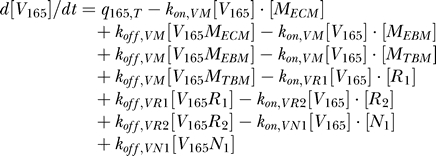


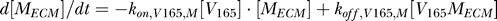


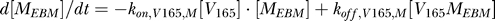


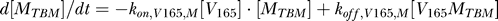


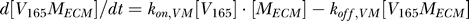


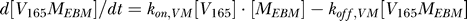


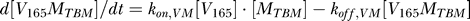
Here [*V*] represents the concentration of a VEGF isoform; [*M*] the concentration of VEGF binding sites in the ECM, EBM, or TBM; [*R*] the density of cell surface receptor tyrosine kinases; [*N*] the density of cell surface Neuropilin; *q_V,T_* is the secretion rate of VEGF from the tumor cells, and *k_on_* and *k_off_* are the kinetic rates of binding and unbinding, respectively. Although we note VEGF as secreted from the tumor cells, VEGF secreted from other sources, e.g., endothelial cells themselves, if significant, could be included in the same term by adding all the sources of VEGF together. In addition, VEGF secretion is assumed constant; no feedback mechanism is included for increasing or decreasing secretion in response to VEGF receptor signaling. Such a mechanism could serve to blunt the response of each of the inhibitors. For cell surface receptors, we have the following equations:

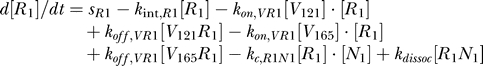


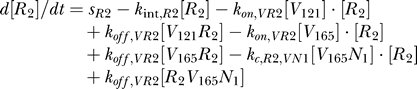


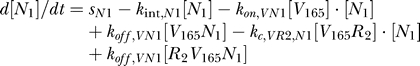


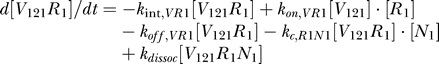


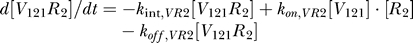


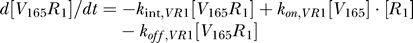


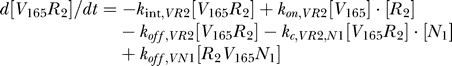


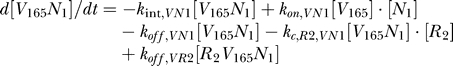


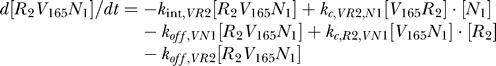


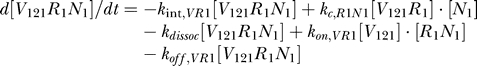


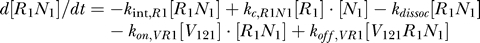
where [*VR*] is the concentration of VEGF-bound receptor; [*VN*] is the concentration of VEGF-bound Neuropilin; [*RVN*] is the concentration of the VEGF-coupled VEGFR–Neuropilin complex; *k_c_* is the coupling rate of Neuropilin-bound or VEGFR-bound VEGF to a second receptor; *s_R_* is the insertion rate of new receptors into the membrane, and *k_int_* is the internalization rate of receptors.

**Figure 2:**
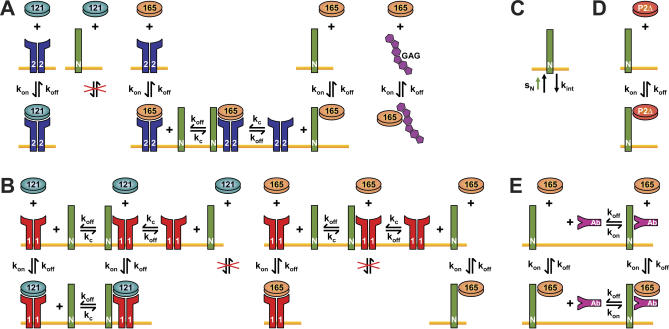
Interactions between VEGF_121_, VEGF_165_, and Their Cell Surface Receptors VEGFR1, VEGFR2, and Neuropilin-1, and Interstitial GAG Binding Sites (A) VEGF_121_ binds to VEGFR2 but not Neuropilin-1. VEGF_165_ binds both receptors as well as GAG chains in the interstitial space. VEGF_165_ bound to Neuropilin-1 can diffuse laterally on the cell membrane and bind VEGFR2 (and vice versa), coupling these receptors together, even though the receptors themselves do not interact. (B) VEGF_121_ and VEGF_165_ both bind VEGFR1. Neuropilin-1 and VEGFR1 interact directly, forming a complex that is permissive for VEGF_121_ binding but not VEGF_165_. (C) Inhibition of Neuropilin-1 expression results in a decrease in the insertion rate of Neuropilin receptors into the cell membrane (s_N_). (D) PlGF_2Δ_, a fragment of placental growth factor, competes with VEGF_165_ for the binding site on Neuropilin-1. (E) An antibody to Neuropilin-1 that does not interfere with VEGF_165_ binding can block the coupling of VEGF_165_–Neuropilin to VEGFR2, resulting in sequestration of VEGF on nonsignaling Neuropilin.

The signaling ligand–receptor complexes formed by VEGF_121_, VEGF_165_, and their receptors are shown in Figure 1, and the full system of interactions is shown in Figure 2A and 2B. The coupled set of ordinary differential equations is solved for appropriate values of the parameters (below) to find the initial steady state concentrations of all molecular species. Then, an intervention takes place and the system of equations is simulated to find the response for 48 hours following the intervention.

#### Therapeutic interventions.

One of three Neuropilin-targeting therapies is used and the system is observed for 48 hours. The first modification of Neuropilin expression is a change in the value of s_N_, the insertion rate of Neuropilin into the membrane (Figure 2C). No other parameters are affected, and no new molecular species, interactions, or equations are required. The second and third interventions require the addition of two new molecular species: PlGF_2_ (or a fragment thereof) and an antibody to Neuropilin (Ab_NRP_) or a molecule-blocking Neuropilin–VEGFR2 coupling in a similar fashion, and their associated interactions.

As described in the introduction, PlGF_2Δ_ is a fragment of placental growth factor that binds only to the VEGF_165_ binding sites of Neuropilin (Figure 2D). This is the only added interaction required for simulation of this therapy. The addition of this interaction requires two additional model equations (for PlGF_2Δ_ and the PlGF_2Δ_–Neuropilin complex):

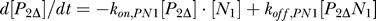


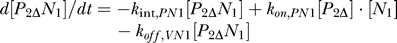
It also requires modifications to the equation governing Neuropilin concentration:

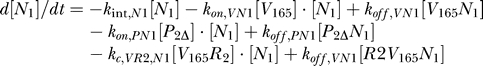
The kinetics of the interaction were obtained from simulations of in vitro competition experiments [9,14]. We assume that this protein does not bind to the ECM (although the full-length PlGF_2_ may bind). ECM binding would further reduce its predicted efficacy.

The antibody to Neuropilin being investigated here does not affect VEGF binding to Neuropilin [9,20–23]. It binds to a different domain of Neuropilin and allows VEGF binding to proceed; however, it then prevents the coupling of VEGFR2 to the VEGF–Neuropilin complex (Figure 2E). The binding of the antibody to Neuropilin, as well as the binding of VEGF_165_ to the antibody-bound receptor, are added to the interactions in the model. New equations for the antibody and the complexes it forms are added to the model:

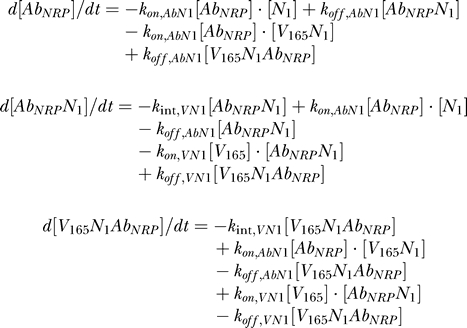
In addition, several equations in the model are modified to include the effects of the antibody:

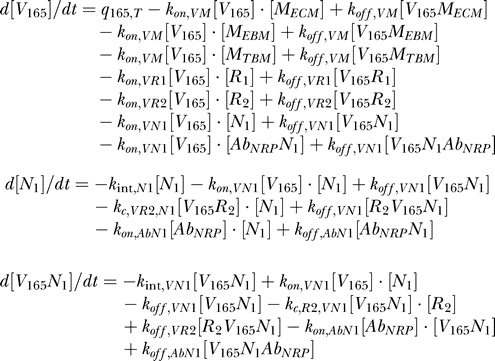



#### Parameters.

The parameters required for simulation of this model fall into three categories: geometric, kinetic rates, and initial concentrations; they are given in Tables 1–3.

**Table 1:**
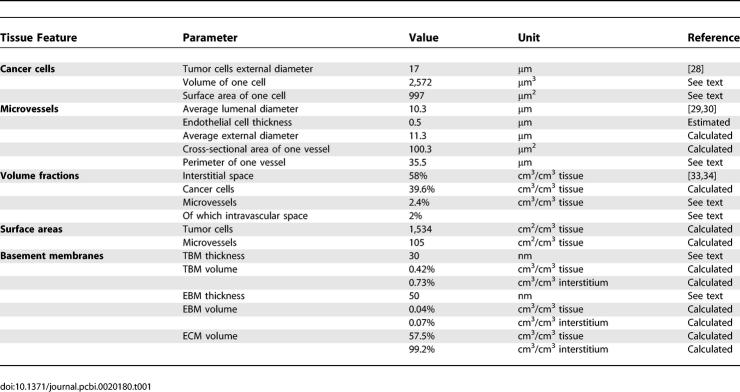
Microgeometrical Parameters for Breast Cancer

**Table 2:**
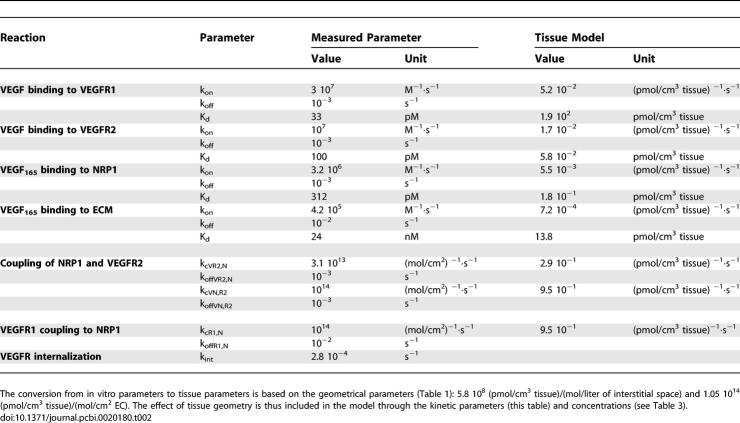
Kinetic Parameters of VEGF System

**Table 3:**
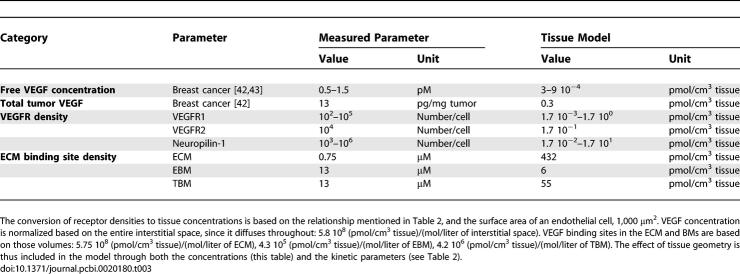
VEGF Concentration and VEGFR Density

#### Geometry.

The model is applicable to any solid tumor. To be specific, in this study we use the geometric parameters typical for breast cancer as summarized in Table 1. The tumor cells are assumed to have the same volume as the MCF7 breast tumor cell line, which have a mean diameter of 17 μm [28], for an equivalent spherical volume of 2,572 μm^3^. A sphere of this volume would have a surface area of 908 μm^2^, but tumor cells are not spherical. We assume a dodecahedral cell of the same volume, which has a surface area of 997 μm^2^ (10% increase over the sphere). The average diameter of capillaries in breast cancer has been measured as 10.3 μm (lumenal diameter) [29,30]. Assuming an endothelial cell thickness of 0.5 μm, this would yield a cylindrical cross-sectional area of 100 μm^2^ and an outer perimeter of 35.5 μm. However, microvessels are not cylindrical, and to find the true perimeter we used a relationship between total perimeter and total cross-sectional area measured in breast cancer. The increase in perimeter over that predicted by cylinders from the area data is 23% [31,32], for a capillary perimeter of 43.7 μm.

The average extracellular fluid volume in breast tumors has been measured from 51%–63% [33,34], although the range of individual measurements is even wider. We assume a value of 60%, which is divided into interstitial space and vascular space. The remainder of the tissue is intracellular fluid contained in the tumor cells themselves, as well as in the endothelial cells of the blood vessels.

The vascular density appears to range widely for breast cancer, with 100–500 capillaries/mm^2^ cross-sectional area of tissue measured in different tumor samples [35]. Many studies of vascular density in breast cancer have been performed (see [36] for a comprehensive list and review), and typical average values are 100–250 capillaries/mm^2^. For the capillary dimensions described above, a capillary density of 235 capillaries/mm^2^ gives a vascular volume of 2% cm^3^/cm^3^ tissue. This is lower than the microvascular volume of 5% (even allowing for volume of the endothelial cells) found in studies of vascular volume [37,38]. This volume would require a significantly higher capillary density (490 capillaries/mm^2^) for the average capillary size noted above. The reason for the discrepancy may be the inclusion in that study of larger microvessels, or the differences between the types of cancer studied. We performed most of the simulations (Figures 3–7) using the 2% vascular space, but also performed simulations for the larger vascular volume and included those for comparison (Figures 8 and 9).

**Figure 3:**
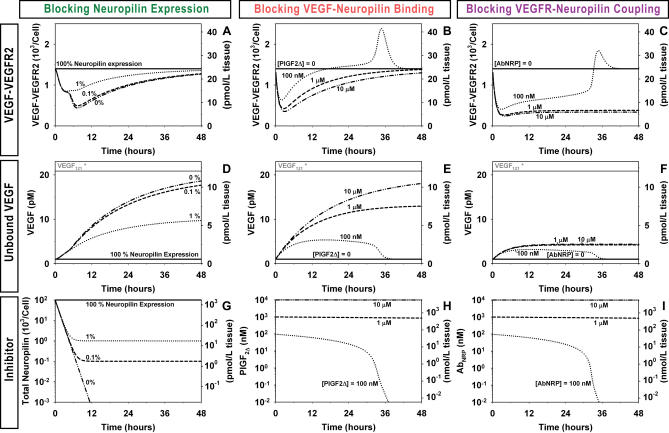
VEGF Binding to VEGFR2 In Vivo Is Inhibited by Targeting Neuropilin-1 (A–C) The time course of VEGF–VEGFR2 complex formation on the endothelial cells following each of the three treatments. For blocking VEGF–Neuropilin-1 binding (B) and VEGFR–Neuropilin-1 coupling (C), this figure represents bolus intratissue protein delivery at time 0. For Neuropilin-1 expression blockade, insertion of Neuropilin-1 into the membrane decreases to the indicated level at time 0. The VEGF_121_–VEGFR2 binding curve is indistinguishable from the no-treatment line in each case. The tumor modeled here expresses 10,000 VEGFR2 and 100,000 Neuropilin per endothelial cell. (D–F) Free (unbound) VEGF concentration in the interstitial space. *VEGF_121_ secreted at the same rate as VEGF_165_. (G–I) The concentration of VEGF inhibitor in the interstitial space, or density of Neuropilin, on the blood vessel endothelial cell surface.

The 2% vascular volume gives us an interstitial volume of 58% cm^3^/cm^3^, and having fixed the total vascular volume and the size of each vessel, the volume taken up by the endothelial cells of the microvessels is determined to be 0.4% cm^3^/cm^3^ based on 0.5 μm thickness. The remaining tissue volume (39.6%) is occupied by the cancer cells.

From the relative volumes of each of the extracellular regions, and the surface areas of the vessels and tumor cells, we can calculate the total surface area of all vessels and tumor cells per unit volume of tissue. For the above parameters, they are 105 cm^2^ endothelial cell surface / cm^3^ tissue and 1,534 cm^2^ tumor cell surface / cm^3^ tissue.

Last, both the vessels and the tumor cells have associated basement membranes. The thickness of these was not available for breast cancer, and we have assumed thickness of 50 nm and 30 nm, which are within the range of thicknesses of basement membranes observed in other tissues [39,40], but relatively thin, in consideration of the high density of proteases present in tumors. These thicknesses are used to calculate the volumes of each type of basement membrane in the tumor tissue.

**Figure 4:**
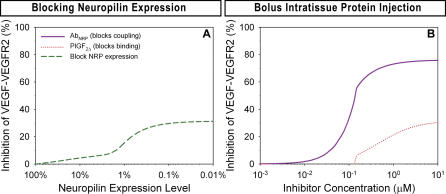
Average VEGF–VEGFR2 Signaling Inhibition The average inhibition of VEGF–VEGFR2 complex formation over the 48 hours following blockade of Neuropilin-1 expression (A), or bolus intratissue protein delivery (B) of competitive binding inhibitor or VEGFR–Neuropilin-1 coupling blocker. Endothelial cells expressing 10,000 VEGFR2 and 100,000 Neuropilin-1.

#### Kinetics.

The kinetic rates for VEGF isoform binding to and unbinding from VEGF receptors and Neuropilins are based on experimental measurements and are similar to those used in previous models [9], and are summarized in Table 2. The ECM binding sites for VEGF are a diverse group of protein and proteoglycan with different affinities. An effective binding affinity is used for the ensemble of sites. VEGF binding affinity to the GAG chains on the proteoglycans of the ECM chains is of a similar order to that of bFGF, and therefore we use similar binding site density and affinity [41]. The affinity of the PlGF_2Δ_ fragment for Neuropilin is assumed to be the same as VEGF for Neuropilin. The affinity of the anti-Neuropilin antibody for Neuropilin has not been measured and is assumed to be 0.1 nM, typical of other antibodies.

**Figure 5:**
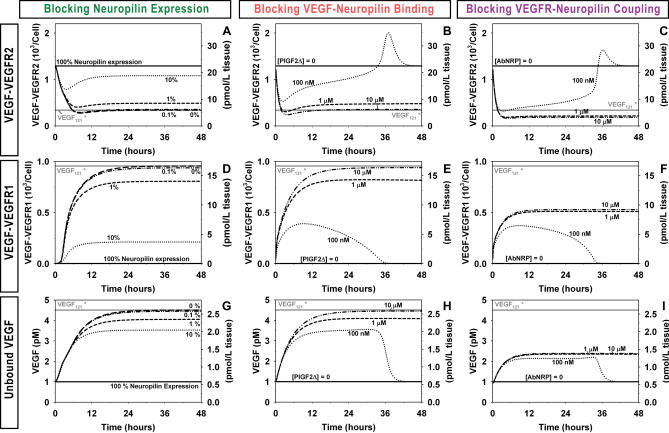
Tissue Expressing High VEGFR1 Levels Responds Differently to Treatment (A–C) Formation of VEGF–VEGFR2 complexes over time following anti-Neuropilin treatment, for a tumor expressing 10,000 VEGFR1 per endothelial cell in addition to the VEGFR2 and Neuropilin-1 expression of Figures 3 and 4. (D–F) VEGF–VEGFR1 complex formation. (G–I) Free (unbound) interstitial VEGF concentration. *VEGF_121_ secreted at the same rate as VEGF_165_.

#### Concentrations.

The initial concentration of unbound VEGF has been determined by microdialysis of breast tumors to be in the range 20–70 pg/ml, or 0.5–1.5 pM [42,43]. This is approximately 10-fold higher than normal breast tissue [44]. We begin all simulations at a steady state with a free VEGF concentration of 1 pM. Although VEGFR1, VEGFR2, and Neuropilin all have been identified as being expressed on the endothelial cells of the vasculature, no quantitative estimate of the receptor density in breast cancer has been published. As a result, we will examine several expression levels of these receptors—starting with estimates from other tissues—to estimate the effect of receptor density on the efficacy of the therapies. For Figures 3, 4, 8, and 9, 10,000 VEGFR2 and 100,000 NRP1 are expressed on each endothelial cell. For Figures 5 and 6, 10,000 VEGFR1 are added. In Figure 7, the sensitivity of the results to VEGFR expression is explored.

**Figure 6:**
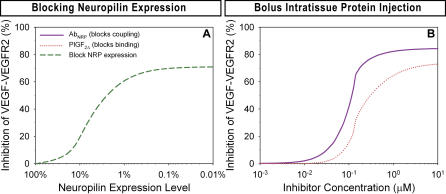
Average VEGF–VEGFR2 Signaling Inhibition over the 48 Hours Following Treatment Endothelial cells expressing 10,000 VEGFR1, 10,000 VEGFR2, and 100,000 Neuropilin-1.

#### Geometrical conversion of the parameters.

For the set of ordinary differential equations above, concentrations are expressed in per-tissue-volume units. That is, interstitial concentrations are expressed as pmol/(cm^3^ tissue) rather than pM; pM in this case would be equivalent to pmol/(liter of interstitium). Surface concentrations are similarly expressed as pmol/(cm^3^ tissue) rather than per-surface-area units such as molecules/cell or pmol/(cm^2^ cell surface area). All of these units may be interconverted using the tissue's characteristic geometrical parameters as described in the legend of Table 3.

For the equations to hold, the units of the parameters used in the equations must also be changed to be consistent, e.g., the units of *k_on_* are converted from (M^−1^ s^−1^) to (pmol^−1^ (cm^3^ tissue) s^−1^) using the fractional volume of the interstitial space. This is explicitly performed in Table 2. Note that the tumor cell surface area, the vasculature surface area, and the interstitial volume all are central to the conversions, and thus changes to the geometry of the tissue (i.e., to these geometrical parameters) will result in changes to the effective kinetic parameters, and to the concentrations of VEGF and the complexes it forms with GAG chains or VEGF receptors.

**Figure 7:**
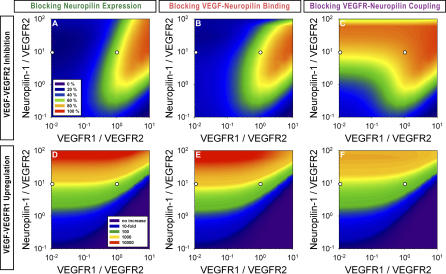
Tissue Specificity of Neuropilin-Targeted Inhibition of VEGF Signaling Tissues that express low levels of Neuropilin-1 are insensitive to all Neuropilin-targeting treatments. The inhibition of VEGF–VEGFR2 signaling is directly proportional to Neuropilin-1 density (A–C), except at very high Neuropilin levels, which can overcome the inhibition. Tissues that express intermediate and high levels of Neuropilin-1 are further distinguished by the level of expression of VEGFR1. Blocking VEGFR–Neuropilin coupling is the most effective treatment to reduce VEGF–VEGFR2 signaling for tissues with any VEGFR1 expression level. However, in high VEGFR1 tissues, the other treatments are also quite effective. All three treatments significantly induce VEGF–VEGFR1 complex formation (D–F). The circles in each figure denote the conditions for Figures 3 and 4 (left, VEGFR1 actually zero in simulations) and Figures 5 and 6 (right) to compare the efficacy of the treatments for different tumors. The results shown are for a Neuropilin-1 expression knockdown to 1%, for 1 μM PlGF_2Δ_, and 1 μM Ab_NRP_.

### Results of Computational Model Simulations

#### Simulations of VEGFR2 and Neuropilin coexpression.

Simulations of VEGF transport in breast cancer, and of therapeutic interventions to inhibit signaling, were developed as described in the Methods section. The simulations are first presented with the endothelial cells of the blood vessels expressing VEGFR2 and Neuropilin but not VEGFR1. We will then examine the impact of VEGFR1 co-expression.

#### Blocking Neuropilin expression.

Blockade of Neuropilin-1 synthesis results in a gradual decline in Neuropilin expression on the endothelial cell surface (Figure 3G) as it is internalized but not replaced by newly synthesized receptors. It reaches a new steady state depending on the level of inhibition. This decrease in Neuropilin expression results in a transient decrease in VEGF–VEGFR2 formation (Figure 3A). The peak inhibition of VEGF–VEGFR2 is dependent on the magnitude of the decrease in Neuropilin-1 expression (and on the initial Neuropilin expression), but for this set of parameters is maximal at ∼60% for complete knockdown. The effect is transient, however, because the loss of Neuropilin reduces the binding of VEGF_165_ to the VEGFR2 expressed on the endothelial cells (Neuropilin has the effect of increasing the amount of VEGF_165_ bound at a given extracellular VEGF concentration). Less VEGF bound means less VEGF internalized, and so the concentration of VEGF in the interstitial space begins to increase as a result of the loss of Neuropilin-1 (Figure 3D). This increased interstitial VEGF concentration results in increased VEGFR2 binding and signaling. The system returns to a new “set point,” with a higher VEGF interstitial concentration compensating for the loss of Neuropilin. Thus, the long-term effect of total Neuropilin-1 loss is that VEGF_165_ behaves just as VEGF_121_ would if it were secreted at the same rate (Figure 3D). VEGF_121_ and its signaling are not affected by any of these treatments, and the formation of VEGF_121_–VEGFR2 complexes is in each case the same as for VEGF_165_ with no treatment.

**Figure 8:**
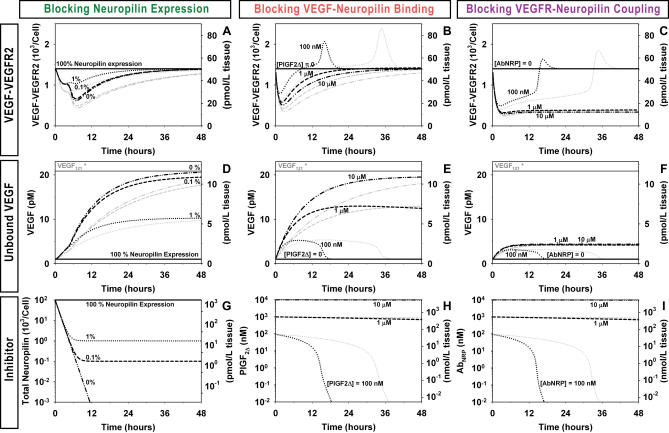
VEGF Signaling Inhibition Is Effective for a Shorter Period of Time for a Tissue with a Higher Microvascular Density (A–C) VEGF–VEGFR2 complex formation on the endothelial cells following each of the three treatments. The tumor modeled here expresses 10,000 VEGFR2 and 100,000 Neuropilin per endothelial cell. Gray lines represent the case of 2% vascular volume, as depicted in Figure 3; the black lines represent 4.2% vascular volume. Note that while the 10^3^/cell, pM, and nM scales apply to both the gray and black lines, the pmol/L tissue scales apply only to the black lines; the normalization is different for the gray lines (see Figure 3 for the correct scales). (D–F) Free (unbound) VEGF concentration in the interstitial space. *VEGF_121_ secreted at the same rate as VEGF_165_. (G–I) The concentration of VEGF inhibitor in the interstitial space, or density of Neuropilin on the blood vessel endothelial cell surface.

#### Blocking VEGF–Neuropilin binding.

Blockade of VEGF–Neuropilin binding again results in a transient decrease in the binding of VEGF_165_ to VEGFR2 (Figure 3B). A peak is reached of 50% inhibition at 10 μM of the protein that blocks VEGF binding. As for Neuropilin expression decrease, this loss of VEGF binding and internalization leads to the buildup of VEGF in the interstitial space (Figure 3E) and results in the loss of the VEGF signaling inhibition. The initial concentration of the inhibitor (PlGF_2Δ_) affects the transient. Note that for higher initial concentrations of the inhibitor (1–10 ΔM), where the concentration of the inhibitor remains essentially constant (Figure 3H), a constant inhibition of VEGF signaling is not obtained. The inhibitor becomes ineffective because the VEGF interstitial concentration increases, and therefore the VEGF binding to VEGFR2 returns to its original level. A low concentration (100 nM) results in depletion of the inhibitor through internalization after 36 hours (Figure 3H). By the time the inhibitor is depleted, and Neuropilin is available for binding again, VEGF concentration has increased substantially (Figure 3E), and this results in a transient overshoot in the binding of VEGF to VEGFR2 (Figure 3B). Thus, signaling is transiently *increased* as the system returns to its previous state, with VEGF concentration declining to its original level (Figure 3E). This is not observed in the Neuropilin expression blocking treatment as the expression level remains low for the 48-hour duration of the experiment.

**Figure 9:**
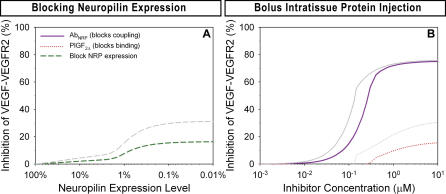
Average VEGF Signaling for the 48 Hours Following Treatment Inhibition Is Decreased at Higher Microvascular Density The gray lines represent the case of 2% vascular volume (as depicted in Figure 4); the colored lines represent a 4.2% vascular volume. The peak inhibition due to the blockade of VEGFR–Neuropilin coupling is the same for both vascular densities; however, the peak for the other two treatments is lower. Endothelial cells expressing 10,000 VEGFR2 and 100,000 Neuropilin-1.

#### Blocking VEGFR–Neuropilin coupling.

Blockade of VEGFR–Neuropilin coupling results in significantly higher peak inhibition than the first two treatments: 80% at 1 μM of the Neuropilin-1 antibody (Figure 3C). In addition, the inhibition is not transient, but sustained over the 48 hours of the analysis, as long as the inhibitor is present in the interstitial space (Figure 3I). For lower initial concentrations of the inhibitor, the antibody is internalized and depleted (after 32 hours), and the system returns to its original signaling state at the same time (Figure 3C, 3F, 3I). There is a slight increase in interstitial VEGF concentration in the presence of the inhibitor (Figure 3F). This is due to decreased internalization of VEGF as a result of lower total binding to VEGFR2 and Neuropilin as result of the loss of coupling (VEGFR2–Neuropilin coupling stabilizes VEGF_165_ association with the cell surface). This increase in VEGF concentration results in the transient overshoot observed in VEGF signaling when the inhibitor is depleted (Figure 3C).

#### Summary of inhibition of VEGFR2 signaling.

Our computer simulations predict that the maximal average inhibition of VEGF–VEGFR2 complex formation over the 48 hours is less than 30% for Neuropilin-1 expression blocking or VEGF binding blocking, but close to 80% for blocking VEGFR–Neuropilin coupling (Figure 4). The blocking of coupling is predicted to be particularly effective in inhibiting VEGF signaling in vivo because Neuropilin-1 can still bind VEGF on the surface of the endothelial cell. Other methods of blocking Neuropilin-1 result in the functional absence of this pro-angiogenic co-receptor; by blocking coupling, however, we have turned Neuropilin-1 into an anti-angiogenic co-receptor, sequestering VEGF on the surface away from the signaling receptors.

While the blockade of VEGFR–Neuropilin coupling exhibits prolonged inhibition of VEGF–VEGFR2 binding (Figure 3C) compared with the other treatments (Figure 3A and 3B), it does not automatically follow that this extended decrease in signaling will result in increased inhibition of angiogenesis and vascular growth, as timing of VEGF signaling, as well as other cytokines, may play a role.

#### Effect of high VEGFR1 expression.

Some tissues, but not all, also demonstrate VEGFR1 expression on endothelial cells. Unlike VEGFR2, VEGFR1 interacts directly with Neuropilin-1, and the complex formed can bind VEGF_121_ but not VEGF_165_ [45,46] (Figure 1B). Thus, the presence of Neuropilin-1 diverts VEGF_165_ from binding VEGFR1 to binding VEGFR2. In addition, VEGFR1 itself transduces VEGF signals, though it appears that these may inhibit or modulate VEGFR2 signaling [47]. Thus Neuropilin-1 increases VEGFR2 signaling both by presentation of VEGF_165_ and by inhibiting binding of VEGF_165_ to VEGFR1. Since both of these processes would be inhibited by blocking Neuropilin-1, we analyzed a tissue that expressed high levels of VEGFR1 to determine the effects of these treatments.

All three of the treatments now resulted in significant, sustained decreases in VEGF_165_−VEGFR2 signaling (Figure 5A–5C). This is primarily due to the binding of VEGF_165_ to VEGFR1, which was not possible while Neuropilin-1 was forming complexes with VEGFR1. With the Neuropilin-1 decreased or occupied, there is increased formation of VEGF–VEGFR1 complexes (Figure 5D–5F). The inhibition of VEGFR2 signaling that results from blocking Neuropilin-1 expression or VEGF binding in these tumors is not transient, as the internalization of VEGF by VEGFR1 prevents interstitial VEGF buildup as significant as that in the absence of VEGFR1 (compare Figure 5G–5I with Figure 3D–3F).

The result of functional Neuropilin-1 loss (by expression or binding blockade) is, as before, that VEGF_165_ signaling becomes similar to VEGF_121_ signaling. In tissues that express VEGFR1 (with or without Neuropilin), most VEGF_121_ binds to VEGFR1 (higher affinity than VEGFR2), and, thus, the VEGF_121_ signaling through VEGFR2 is decreased as the receptors compete for the available ligand. Blockade of VEGFR-Neuropilin coupling, on the other hand, results in a decrease in VEGFR2 signaling beyond that of VEGF_121_ (Figure 5C). In addition to blocking VEGFR1-Neuropilin coupling, it results in the sequestration of VEGF_165_ by the nonsignaling Neuropilin-1. Thus, the model still predicts that for tissues expressing high levels of VEGFR1 on blood vessels, blocking coupling will be the most effective therapy, though not by as great a margin (Figure 6).

#### Tumor specificity: Variation in receptor expression.

The vasculature in different tissues—and in different tumors—expresses different amounts of each VEGF receptor. This can result in differing responses to therapies. The tissue-specific nature of these interactions is investigated by varying the receptor expression of both Neuropilin-1 and VEGFR1 (Figure 7). The inhibition of VEGFR2 signaling by each of the three treatments (Figure 7A–7C) is directly proportional to Neuropilin-1 expression, and tissues that express only low levels of Neuropilin-1 are predicted to be insensitive to these treatments. Only at extremely high levels of Neuropilin (above 10^6^ per cell) can the vessels overcome the inhibition and maintain functional involvement of Neuropilin in VEGFR-signaling enhancement. VEGFR–Neuropilin coupling blockade is the most effective treatment for all tissues (Figure 7C), though in tissues expressing high VEGFR1 on the vasculature, the other treatments are also predicted to be quite effective. Thus, it is essential to understand the microenvironment of the tissue of action of the drug being administered, as a drug that efficiently decreases VEGF signaling in one tissue may not do so in another. In particular, a method to measure the level of receptor expression on cell surfaces in vivo would be helpful in determining which tissues are suitable for which treatment.

It is important to note that for all endothelial cell types that have been measured, from various different tissues, cell surface VEGFR1 expression is equal to or less than VEGFR2 expression, which suggests that blocking receptor coupling will be the most effective treatment.

VEGF–VEGFR1 binding is increased by each Neuropilin treatment except for tissues expressing a high level of VEGFR1 and a low level of Neuropilin-1 (Figure 7D–7F). In these tissues, most Neuropilin is predicted to be normally bound to VEGFR1, but there is excess VEGFR1, and blocking the Neuropilin–VEGFR1 interaction does not substantially increase the VEGF–VEGFR1 binding.

#### Larger vascular volume in tumor.

The vascular volume varies significantly from tumor to tumor. Here we simulate a tissue in which the vessels occupy 5% of the tissue volume (the vascular space 4.2% of volume). For this simulation, the vessel surface area is 218 cm^2^/cm^3^, and the volume of the interstitial compartment is reduced slightly. The increased vascularity also increases the total volume of endothelial basement membrane. The increased vessel density results in increased receptor density in the tissue, and so the inhibitors are depleted more quickly (Figure 8H–8I). The peak inhibition due to expression or binding blockade is lower and more transient than before (Figure 8A–8B). The average inhibition over the 48 hours of the simulation is lowered for all treatments, except for high concentrations of the coupling inhibitor (Figure 9).

## Discussion

The vasculature that invests a tumor has been recognized as a therapeutic target that can be exploited to starve the tumor or to increase the delivery of drugs directly to the tumor [48]; vascular density is a negative prognostic factor for many cancers, including breast cancer [36]. The VEGF family of growth factors, and their receptors, are involved in the process of angiogenic vessel growth in tumors, and targeting these molecules may prove to be a successful anti-cancer therapy.

We have previously built a computational model that describes the behavior of the VEGF–VEGF receptor system [9,13] and agrees with all available in vitro experimental literature [10,14–16]. We have extended that model here to simulation of an in vivo tissue to investigate the signaling of VEGF through blood vessel endothelial cell surface receptors in cancer. The model is generally applicable to most cancers, given appropriate values for the parameters. Here the model is applied to human breast cancer and the impact of several treatments that target Neuropilin-1 to inhibit VEGF_165_ signaling. One of these therapeutic strategies, blockade of VEGF receptor–Neuropilin coupling on the cell surface, is predicted to be significantly more effective and more persistent than the others at all concentrations and for all tissues studied. The effectiveness of this antibody to inhibit pathological angiogenesis has been validated experimentally [15]. In that case, the antibody inhibited neovascularization in the eye, but our simulations predict that the antibody would also be effective as an anti-tumor therapy, though this has yet to be tested in vivo.

Thus, the model shows that it is not enough to identify Neuropilin as a therapeutic target due to its specificity for VEGF_165_; different methods of targeting Neuropilin result in different outcomes.

It should be noted that here we define effectiveness as the decrease in VEGF–VEGFR2 binding over the 48 hours following treatment. Timing is a crucial component of intracellular signaling, and it is not clear at this point whether the prolonged inhibition of signaling (e.g., Figure 3C) is more effective at inhibiting angiogenesis and vascular growth than transient inhibition (e.g., Figure 3A–3B). In addition, other cytokines could partly compensate for the loss of VEGF–VEGFR2 signaling.

For VEGFR2, Neuropilin-1-expressing vasculature, inhibition of VEGF–Neuropilin-1 binding, or Neuropilin-1 expression blockade result in transient VEGFR2-signaling decrease, even though the inhibiting factor is not transient. Sustained high levels of the VEGF–Neuropilin-1 binding inhibitor (or sustained depletion of Neuropilin from the surface) in the interstitial space result in VEGF–VEGFR2 signaling recovering from the inhibition during the simulation due to the increasing interstitial VEGF concentration.

The vasculature in different types of breast cancer expresses different levels of VEGFR1, VEGFR2, and Neuropilin-1. In fact, the expression of these receptors may vary spatially within a tumor [49]. The effectiveness of each treatment is predicted to be directly dependent on the receptor expression profile of the target tissue. Increased Neuropilin results in increased efficacy of anti-Neuropilin therapy, except at very high Neuropilin levels, at which the inhibition becomes ineffective. VEGFR1 coupled to Neuropilin does not sequester VEGF_165_, and so the presence of VEGFR1 affects the outcome of therapeutic interventions significantly. For application to other cancers, different geometries would hold, and different expression levels of the receptors. However, the basic molecular interactions would be the same, and the qualitative conclusions of our study can be generalized to other tumor types.

We have not included the effect of VEGF receptors on the tumor cells themselves. This is increasingly being identified as a source of autocrine survival and growth signaling for the tumor cells. We focused instead on the impact on pro-angiogenic signaling at the endothelial cell surface. Inclusion of VEGF receptors on the tumor cells would not qualitatively change our results. For example, the presence of Neuropilin on tumor cells would offer an alternate route for VEGF (away from the endothelial cells). Blockade of Neuropilin expression, or blocking VEGF–Neuropilin binding, would result in the VEGF that normally binds to tumor cells being redirected to the endothelial cells, possibly increasing pro-angiogenic signaling. By contrast, blockade of VEGFR–Neuropilin coupling would not have this problem, as VEGF_165_ could continue to bind the Neuropilin receptors on tumor cells and would not be displaced.

This model does not include pharmacokinetics—i.e., the route by which the drugs would get to the tumor. It is only concerned with their activity or efficacy once present at the tumor. As such, even for intratumoral injection, we assume that none of the inhibitor is lost to the bloodstream or lymphatics. Significant loss of this type from the interstitial space would decrease the efficacy of each of these inhibitors. In addition, vascular heterogeneity within a tumor is not addressed in this study.

This is the first computational model of VEGF transport in vivo, and the first molecularly detailed model of VEGF inhibition strategies. Models such as this can also be used to investigate other aspects of drug delivery, e.g., dosing and scheduling, as they deal with the site of action of the drug. Testable predictions of this model include the increase in interstitial VEGF concentration in response to the administration of each of the therapeutic strategies, as well as the characteristic signaling inhibition that could be detected at the level of receptor phosphorylation. These predictions should stimulate extensive experimental studies, and the model presented here would serve as a quantitative guide to experimental design.

For example, preclinical models of breast cancer could be used to test the therapeutic strategies and validate the model. Following characterization of breast cancer lines and their induced vasculature, VEGFR2–NRP1 and VEGFR2–VEGFR1–NRP1-expressing candidates would be selected for comparison (Figures 3 and 5). After administration of each of the therapies, the unbound interstitial concentration of VEGF (Figure 3D–3F, Figure 5G–5I) and the phosphorylation level of VEGFR2 (Figure 3A–3C, Figure 5A–5C) would be measured at several time points and compared with controls. Six, 12, 24, 36, and 48 hours appear to be relevant to the dosing used in these simulations. These time points are earlier than typical preclinical endpoints and are an example of how the simulation results can help direct experimental design. In these experimental results, we would expect to observe the characteristically different behavior of each of the therapeutics in VEGFR1-negative breast cancer (Figure 3), and their similarity in VEGFR1-positive breast cancer (Figure 5).

Analogous to the drugs targeting Her2-positive breast cancer that are not effective against Her2-negative breast cancer [50], we demonstrate that the efficacy of some anti-angiogenic strategies will depend on the receptor expression levels on the tumor-associated vasculature. For anti-Neuropilin treatment, this includes not just Neuropilin expression, but also that of its signaling co-receptors VEGFR1 and VEGFR2. Analysis of a system such as this using computational or systems biology tools can provide significant insights into the success and failure of drugs targeting angiogenesis and other processes, and aid in the design and optimization of novel therapeutics. The proteins used in these simulations are not the only proteins or drugs that would inhibit Neuropilin; others may be designed or discovered with similar properties, but the same principles would apply in predicting efficacy.

## Materials and Methods

### Model solution and simulations.

Simulations of VEGF transport in human breast cancer were developed based on the model described in the Results section. The appropriate secretion rate for each simulation was used to achieve a steady state, free (unbound) VEGF concentration of 1pM. Then a therapeutic intervention was simulated by changing appropriate parameters or introducing new molecular species into the system. The complete set of coupled nonlinear ordinary differential equations (see Results) were solved using a Runge–Kutta integration scheme, implemented in Fortran, and run on a desktop PC. The parameters used for these simulations are based on experimental measurements, as detailed in the Results section.

## Supporting Information

### Accession Numbers

Accession numbers from UniProt (http://www.pir.uniprot.org/) are Human VEGF-A (VEGF) (P15692), Human PlGF (P49763), Human VEGFR1 (P17948), Human VEGFR2 (P35968), and Human Neuropilin-1 (NRP1) (O14786).

## Abbreviations

EBM: endothelial cell basement membrane
ECM: extracellular matrix
TBM: tumor cell basement membrane
VEGF: vascular endothelial growth factor
VEGFRs: VEGF receptor tyrosine kinases
